# Effect of Voidage on the Collapsing Bed Dynamics of Fine Particles: A Detailed Region-Wise Study

**DOI:** 10.3390/nano12122019

**Published:** 2022-06-11

**Authors:** Syed Sadiq Ali, Agus Arsad, Kenneth L. Roberts, Mohammad Asif

**Affiliations:** 1 School of Chemical and Energy Engineering, Faculty of Engineering, Universiti Teknologi Malaysia, Johor Bahru 81310, Johor, Malaysia; sadiq.chem179@gmail.com; 2 UTM-MPRC Institute for Oil and Gas, School of Chemical and Energy Engineering, Faculty of Engineering, Universiti Teknologi Malaysia, Johor Bahru 81310, Johor, Malaysia; agus@utm.my; 3 Chemical Engineering Department, College of Engineering and Computing, University of South Carolina, Columbia, SC 29208, USA; ROBERTS0@mailbox.sc.edu; 4 Department of Chemical Engineering, King Saud University, P.O. Box 800, Riyadh 11421, Saudi Arabia

**Keywords:** fluidization, collapse bed, hydrodynamics, bed voidage, purge flow, nanosilica

## Abstract

Bed collapse experiments provide vital information about fluidized bed hydrodynamics. In this study, the region-wise bed collapse dynamics of glass beads, titania (TiO_2_), and hydrophilic nanosilica (SiO_2_) particles with widely different voidages (ε) of 0.38, 0.80, and 0.98, respectively, were carefully investigated. These particles belonged to different Geldart groups and exhibited varied hysteresis phenomena and fluidization indices. The local collapse dynamics in the lower, lower-middle, upper-middle, and upper regions were carefully monitored in addition to the distributor pressure drop to obtain greater insight into the deaeration behavior of the bed. While the collapse dynamics of glass beads revealed high bed homogeneity, the upper middle region controlled the collapse process in the case of titania due to the size-based segregation along the bed height. The segregation behavior was very strong for nanosilica, with the slow settling fine agglomerates in the upper bed regions controlling its collapse dynamics. The collapse time of the upper region was 25 times slower than that of the lower region containing mainly large agglomerates. The spectral analysis confirmed the trend that was observed in the pressure transients. The clear presence of high frequency events at 20 and 40 Hz was observed in the nanosilica due to agglomerate movements. The residual air exiting the plenum was strongly affected by the bed voidage, being lowest for the nanosilica and highest for the glass beads.

## 1. Introduction

Fluidized beds are often preferred over packed beds due to their superior gas–solid contact, higher mass and heat transfer rates, and efficient gas–solid handling and mixing [1,2,3,4,5,6]. However, the fluidization of ultrafine and fine powders belonging to Geldart group C is difficult due to strong inter-particle forces (IPFs), which lead to severe bed non-homogeneities and poor interphase phase mixing [7,8,9,10,11,12,13,14,15,16]. The fluidization of ultrafine nanoparticles can either display agglomerate particulate fluidization (APF) or agglomerate bubbling fluidization (ABF) owing to the large agglomerate formation as a result of the IPFs. Whereas a high minimum fluidization velocity and a low bed expansion are observed in ABF, uniform particulate fluidization is the main characteristic of APF [2,17,18,19,20,21,22,23]. Moreover, the fluidization of ultrafine powders often exhibits the size segregation of agglomerates along the length of the bed. For example, in one case, the ABF behavior of nanosilica led to size-based stratification along the bed height such that agglomerates present in the lower bed region were five to ten times larger than those present in the upper layer [24]. In some cases, agglomerates as large as 2000 µm were found in the lower layer, which were almost ten times larger than those in the upper layer [3]. In view of these challenges, an efficient design and the successful large-scale operation of fluidized beds require a detailed understanding of their hydrodynamics.

An important tool for analyzing the fluidized bed hydrodynamics is the collapse bed study, which can provide meaningful insight into the bed’s dynamic behavior. The fluid flow is instantly stopped in a steady fluidized bed, and the solid particles are allowed free fall under gravity, until the bed attains complete rest [22,25,26,27,28,29,30]. During the first phase of the collapse process, the residual air escapes as bubbles followed by the free fall of the particles. As the particles settle, a stationary layer develops at the bottom, and a dense phase, with moving particles, simultaneously sediments at the top of the layer. The interface of the stationary layer appears to move upwards, while the upper meniscus of the dense phase moves downwards until both coincide to a single surface, thereby marking the end of the collapse process. The pressure gradient gradually reaches zero as the bed attains complete rest [25,31]. The collapse phases are controlled by the IPFs, drag force, and gravitational force, which makes the collapse process an important tool for identifying the dominant forces in the bed.

By monitoring the local pressure drop transients, Asif and Ali [22] noted the occurrence of three prominent events characterizing three different phases of the collapse process. In the first phase, the bed fell rapidly until the pressure transient became zero. With the negative drag increasing with the downward fall of the particles, the pressure drop kept on decreasing, ultimately reaching a maximum negative value marked as the occurrence of the second event. The pressure drop then gradually increased again to a zero value, marking the third event and signaling the end of the collapse process. The bed height declined rapidly in the first phase, while slow solid particle settling occurred in the second phase. The bed height remained stationary in the third phase. The authors compared the time periods of all the phases by varying the initial fluid velocity and found that the total bed settling time did not depend upon the initial superficial velocity. A mathematical model of the bed collapse, proposed Nie and Liu [25], which successfully predicted the height of the interfaces and the pressure drop at different locations along the height of the bed, was used to predict the agglomerate diameter in a collapsing bed of nanosilica [32]. Another work used collapse bed experiments to study the effect of pre-mixing the nanosilica with inert Geldart group A particles as an aid for enhancing the fluidization quality [28].

The deaeration of the residual air during the collapse process critically governs the collapse process. The residual air moves upward during a bed collapse in a conventional fluidized bed [22,28,29,31,33]. However, other deaeration strategies have also been suggested in the literature [24,26,34,35]. Santos et al. [36] added a vent below the fluidized bed, which was active only during the collapse process, and termed this configuration as a double drainage arrangement. Both single (conventional) and double drainage configurations were used with FCC catalysts by the authors. The duration of the bed collapse was substantially lower for the double drainage configuration compared to that of the single drainage deaeration. Owing to the downward movement of the residual air, a negative pressure drop was prominently observed in the lower bed regions for the double drainage configuration. Cherntongchai, Innan [34] also used a modified air passage configuration and proposed a mathematical model for calculating the dense phase voidage and the solid velocity [35]. Lorences, Patience [26] used single drainage, double drainage, and zero drainage configurations to study the collapse dynamics of mixtures of VPO and FCC catalyst particles of different size distributions. While the deaeration durations were identical for the zero and single drainage configurations, shorter deaeration occurred when the double drainage configuration was used. The initial velocity did not affect the collapse process in the author’s research. Ali et al. [24] studied the collapse of a pulsed flow fluidized bed using three different flow strategies, namely single drainage, dual drainage, and modified dual drainage. The square-wave flow pulsations were introduced using a solenoid valve. The modified dual drainage eliminated the initial flow spike at the beginning of the flow when the solenoid valve opened. This strategy reduced the size-based segregation of the nano-agglomerates and resulted in a smoother and faster bed collapse. 

While the investigation of the collapse dynamics in the past mainly focused on the effect of the drainage configuration for the deaeration of the residual gas [24,26,34], the particle classification [17], and the fluidization assistance [27,29], there has not been any focused study on the effect of agglomeration induced size stratification along the bed height, which is a common occurrence in the case of the ultrafine nanoparticles. Any detailed investigation into this effect, however, requires a careful monitoring of the collapse dynamics in the different regions of the bed along its height to clearly delineate the effect of the size stratification of nano-agglomerates. For deeper insights into the collapse behavior, a comparison with other types of particles is also needed. Therefore, three different kinds of powders, viz., glass beads, titania, and ultrafine nanosilica, were selected in this study. We used hydrophilic nanosilica in this study owing to its strong agglomeration behavior that results in ABF behavior [3,24]. Apart from widely different voidages, these powders also significantly differ in their physical properties, and therefore belong to different groups of Geldart’s classification [15]. Note that commercial grade nanosilica and titania find large-scale applications in several industries such as the paint, catalyst manufacturing, and pharmaceutical industries [37,38]. A set of pressure transducers located along the height monitored the local collapse dynamics during the progress of the collapse process in different bed regions of the fluidized bed. The dual drainage configuration for the residual gas deaeration was used. In addition, the distributor pressure drop was also monitored to obtain a better understanding of the gas flow through the distributor during deaeration.

## 2. Experimental

### 2.1. Experimental Set Up

**Figure 1:**
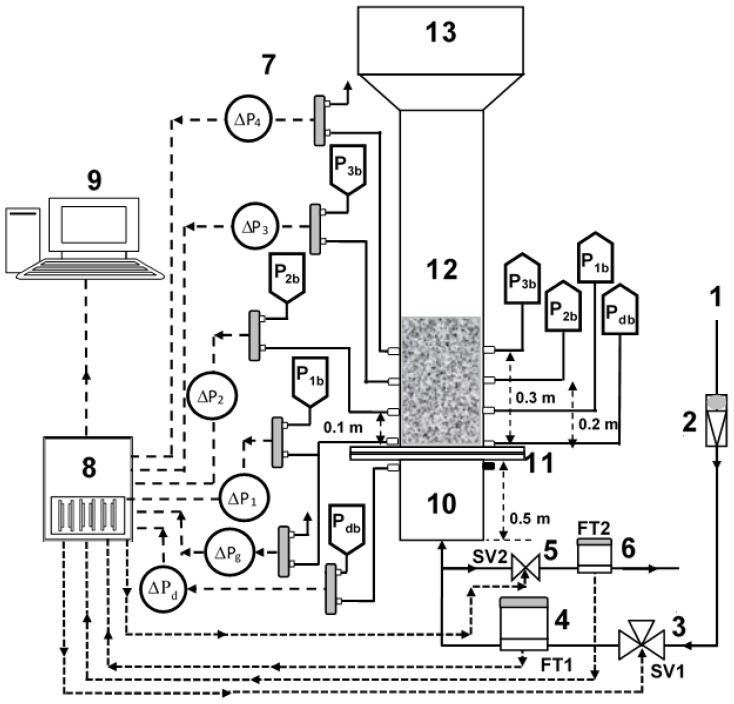
Experimental set-up schematic; (1) Air supply; (2) Flowmeters; (3) 3-way solenoid valve; (4) Flow totalizer; (5) 2-way solenoid valve; (6) Flow totalizer; (7) Pressure transducers; (8) Data acquisition system; (9) Computer; (10) Calming section; (11) Distributor; (12) Test section; (13) Disengagement section.

The experimental set up schematic is shown in Figure 1. The test section was a transparent perplex column, 1.6 m in length and 0.07 m internal diameter. A 0.3 m long plenum, beneath the test section, eliminated inlet disturbances of the fluidizing air. A perforated plate distributor with 2 mm holes in a circular pitch and 2.5% open area was used to separate the test section from the plenum section. Such a distributor design ensured uniform distribution of the inlet gases across the cross-section of the test section and eliminated the dead zones. A 0.14 m diameter and 0.3 m long disengagement section at the top of the test section helped to suppress the elutriation of particles at high fluid velocities. 

The dual drainage deaeration scheme was implemented by allowing another residual air exit from the bottom of the fluidized bed during the bed collapse. This was carried out by using a normally closed 2-way solenoid valve, SV2 (Omega SV 3310, Omega, Norwalk, CT, USA), placed immediately below the plenum chamber. With the start of the collapse process, the solenoid valve was simultaneously energized, thus providing an alternate passage to the escaping residual air from the collapsing bed. As shown in Figure 1, a flow totalizer (FMA-2605A-V2, Omega, Norwalk, CT, USA) was used to measure the flow of the escaping residual air from SV2. 

Several sensitive bidirectional differential pressure transducers (Omega PX163-series, Omega, Norwalk, CT, USA) with a response time of 1-ms and appropriate ranges were used in the experiment. As shown in the figure, the local pressure drop transients in the lower ($∆P_{1}$: 0–10 cm), lower middle ($∆P_{2}$: 10–20 cm), upper middle ($∆P_{3}$: 20–30 cm), and upper regions ($∆P_{4}$: >30 cm above the distributor) were monitored. The global pressure drop ($∆P_{g}$) across the entire bed, and the distributor pressure drop ($∆P_{d}$) across the distributor were also recorded to gain insights into the reverse flow of the exiting residual air through SV2.

A data acquisition (DAQ) system (NI-DAQ-USB-6289) was used to acquire the analog input of the pressure transducers and flow totalizer and control the solenoid valve by using digital output signal. The data sampling rate was set to 100 Hz to capture the high frequency events taking place during the bed collapse.

The segregation tendency was very strong for the nanosilica, with the slow settling of fine agglomerates in the upper bed regions controlling its collapse dynamics, which was five to six times higher than that of glass and titania under similar conditions.

### 2.2. Solid Particles

Three different powders, namely glass beads, titania, and nanosilica, were used in the present work. The physical properties of the particles are listed in Table 1. We used 12 nm ultrafine hydrophilic nanosilica (Aerosil 200, Evonik GmBH, Essen, Germany) with skeletal density of 2200 kg/m^3^. Its median agglomerate size was 12.5 μm (approx.), which was approximately thousand-fold larger than its primary size. The size range of titania was 0.5–90 μm. The particles’ sizes were obtained using Microtrac particle size analyzer (Model S 3500, Montgomeryville, PA, USA).

### 2.3. Methodology

Two different sets of experiments were carried out. In the first set of experiments, the conventional fluidization was carried to determine the minimum fluidization velocity ($U_{mf}$) and fluidization quality (FI) of the different particle samples used. In the second series of experiments, the particles were fluidized at two different fluid superficial velocities ($U_{0}$) of $U_{mf}$ and 2$U_{mf}$ until the steady state fluidization was achieved, and then the airflow to the fluidization column was abruptly interrupted. The purge valve (SV2) was simultaneously opened to provide an alternate passage for residual air. Each set of experiments was performed at least twice to verify the data repeatability. To investigate the effect of height on the collapse behavior, another set of collapse experiments was carried out by adding more material in the column to raise the static bed height by 50%.

## 3. Results

In Section 3.1, the experimental results of the steady-state fluidization are presented for three different solid particles used in this study. The results of the bed collapse study are then considered in Section 3.2.

### 3.1. Conventional Fluidization

**Figure 2:**
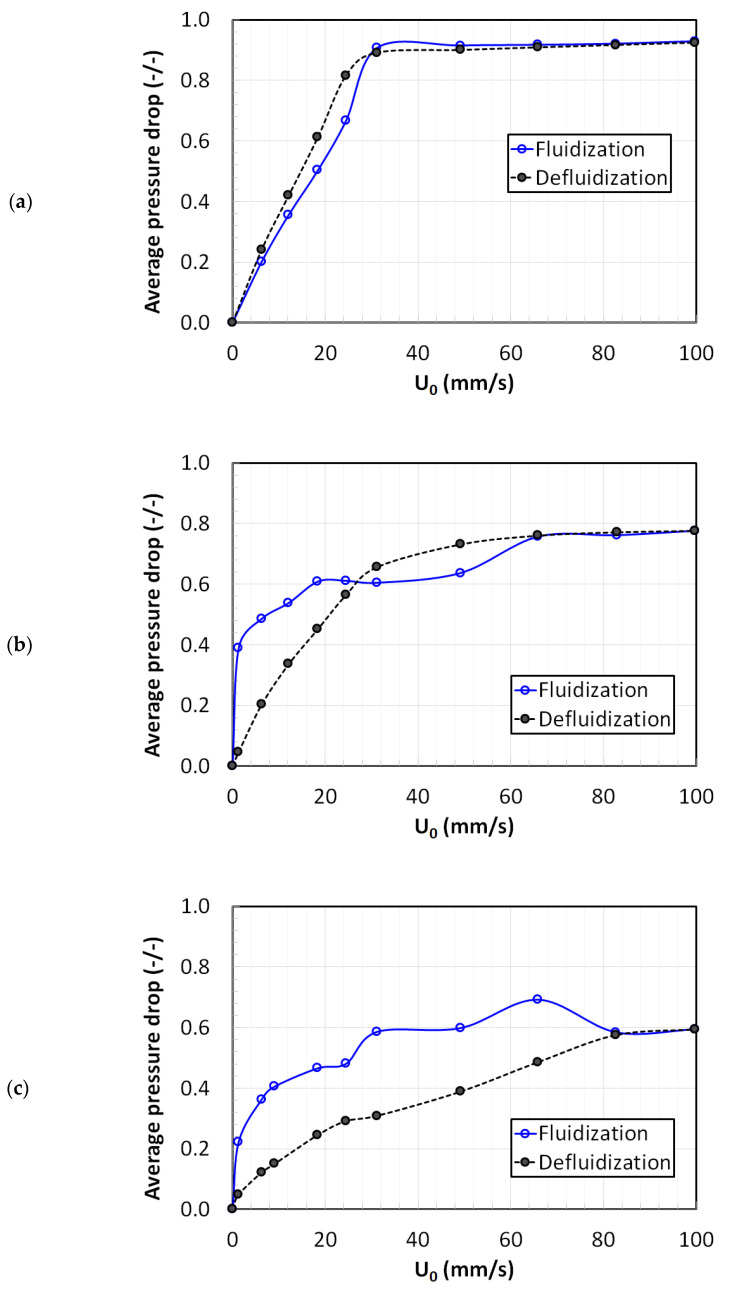
Hysteresis effect between fluidization and defluidization runs for conventional fluidized beds; (**a**) Glass beads; (**b**) Titania; (**c**) Nanosilica.

Figure 2 shows the dependence of the normalized pressure drop on the superficial velocity of the fluidization gas during the conventional fluidization experiments. The pressure drop was normalized by dividing the overall pressure drop across the entire bed $\bar{(∆P})$ by the effective weight of the bed

(1) $$\bar{∆P}=\frac{∆P}{\left(\rho_{b}-\rho_{f}\right)gL}$$

where $\rho_{b},\;\rho_{f}$ are the bulk and fluid densities, respectively, *g* is the gravitational acceleration, and *L* is the bed length across which the pressure drop is measured. Given that the gas density is negligible, the above equation simplifies to

(2) ∆P¯=∆P(mgA)

where *m* is the mass of the particles loaded in the bed, and *A* is the cross-sectional area of the bed. In the literature, the normalized pressure drop is often identified at the fluidization index since it can provide a good indication of the fluidization behavior of the powder [39]. Ideally, the fluidization index should be unity when velocity exceeds the minimum fluidization velocity, which signifies that the pressure drop across the fluidized bed should be equal to the effective bed weight. 

Figure 2a shows the fluidization behavior of glass beads (size: 118 μm), which is categorized as belonging to Geldart’s group A. The flow was first gradually increased up to 100 mm/s, which was more than thrice the $U_{mf}$. The fluidization cycle was followed by a gradual velocity decrease, thus completing the defluidization cycle. An excellent agreement between both the fluidization and defluidization cycles was clearly seen in the figure, thus indicating the absence of the hysteresis and confirming the homogeneity of the bed of glass beads. 

The fluidization behavior of the titania powder is illustrated in Figure 2b. At the lowest air flow, a steep increase in the pressure drop to 0.4 was observed. However, with the further increase in the velocity, the pressure drop increase was gradual and slow before becoming almost constant at 60 mm/s. During the defluidization cycle of the experiment, a gradual velocity decrease showed smooth behavior with the pressure drop almost remaining constant as long as the velocity was greater than 40 mm/s. In this case, the hysteresis phenomenon was clearly evident as the fluidization and defluidization behaviors were significantly different. This behavior was anticipated, given that the titania powder belongs to the group C classification. Ideally, the normalized pressure drop should attain the value of unity when the bed is fully fluidized. For the fluidization of the glass beads, when *U*_0_ ≥ 30 mm/s, the normalized pressure drop was nearly unity (≈0.92). On the contrary, the normalized pressure drop never exceeded 0.76 for the fluidization of titania. This behavior indicates the presence of non-homogeneities caused by the channeling and rat-holes through which the gas bypasses the bed solids, resulting in lower frictional losses. 

Figure 2c shows the fluidization behavior of the highly porous nanosilica powder (ε = 0.98). Silica nanoparticles form agglomerates with a wide size distribution [40]. Their fluidization shows agglomerate bubbling fluidization (ABF) [17]. The bed was composed of three main layers. The upper layer consisted of fine agglomerates while the bottom layer mainly consisted of large and rigid agglomerates. There was a transition layer of different-sized fluffy agglomerates in the middle section of the bed. The size segregation of the nanosilica bed has been reported earlier as well [24,41]. Even at $2U_{mf}$, there was hardly any motion in the lower layer, but a vigorous motion of fine agglomerates was observed in the bed’s upper region. This behavior is an example of a partially fluidized bed and an indication of ABF. The fluidization and defluidization pressure drop profiles showed a substantial difference in this case. While the fluidization cycle displayed a rather unpredictable dependence of the pressure drop on the superficial velocity, a gradual decrease in velocity during the defluidization cycle showed a gradual and smooth pressure drop decrease. A pronounced hysteresis phenomenon was observed in the fluidization of nanosilica. The fluidization index was low, approximately 0.61. Such a low value of the fluidization index was a clear indication of gas channeling and bypassing. The $U_{mf}$ values were calculated from the fluidization curves in Figure 2 and reported in Table 1.

**Figure 3:**
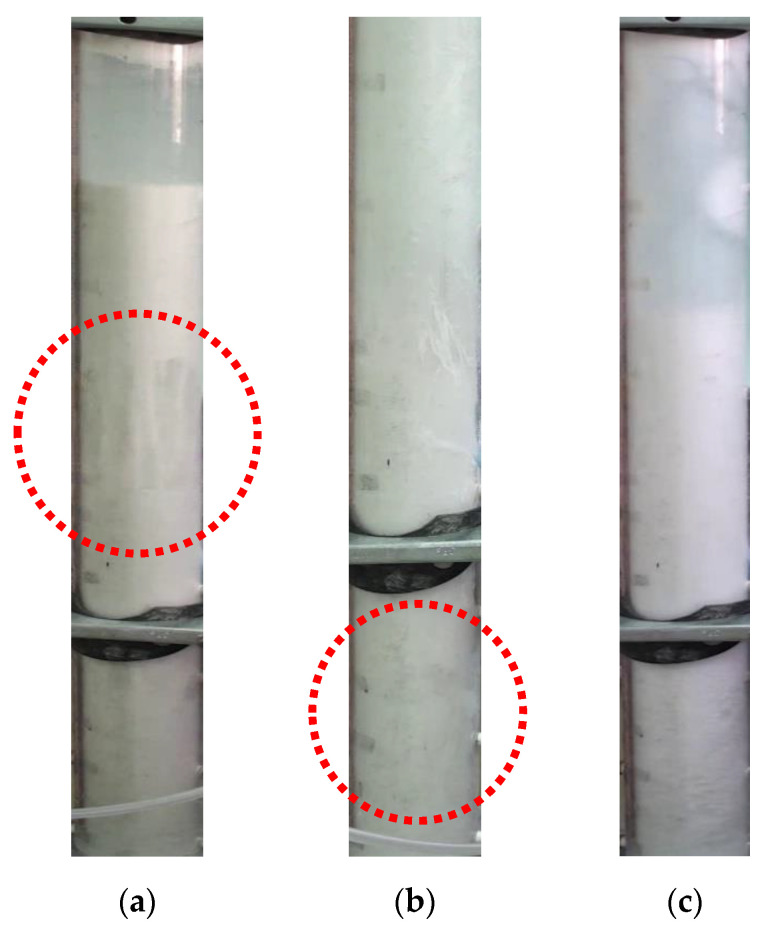
Digital images of solid particles fluidized bed; (**a**) Glass beads; (**b**) Titania; (**c**) Nanosilica.

Figure 3 shows a snapshot of the fluidization behavior of all the three powders. The glass beads displayed bubbling fluidization whereby the air moved as large bubbles through the bed. This is visible in Figure 3a encircled in red. The intensity of the bubbles increased with the increase in the fluid superficial velocity. On the other hand, the titania particles exhibited agglomerate bubbling fluidization (ABF) due to their cohesiveness, whereby the gas bubbles rapidly move upward through the bed [40]. Note that the bubbling behavior of ABF was different from that of the Group A particles, where large air bubbles dominated the fluidization behavior. The bubbles in the titania bed were smaller and irregular and erupted through channels and cavities present in the bed (marked in red in Figure 3b). The expansion was low with a substantial degree of non-homogeneities observed throughout the bed. [17]. As mentioned earlier and seen in Figure 3c, severe size-based segregation was observed during the fluidization of the nanosilica. The large and rigid agglomerates in the lower region hardly showed any movement even at high velocities whereas vigorous motion was observed in the upper layer of the fine agglomerates. The overall bed thus appeared to be only partially fluidized even at high velocities.

### 3.2. Bed Collapse Dynamics

**Figure 4:**
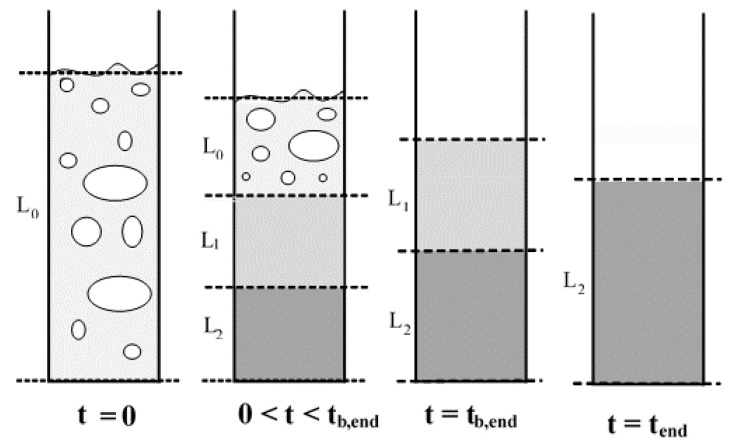
Schematic of the stepwise collapse process of Geldart group A particles.

Figure 4 shows the pictorial representation of the collapse process of Geldart group A particles [24,35]. The collapse occurs in two main stages: a bubbling stage and a sedimentation stage. The bubbles escape in the first stage, while the particles settle under gravity during the second stage. A dense layer ‘$L_{1}$’ is created after the escape of the air bubbles, and a stationary layer of settled particles ‘$L_{2}$’ forms at the bottom of the bed. As more particles progressively settle in the stationary region, the interface between ‘$L_{1}$’ and ‘$L_{2}$’ rapidly moves upwards towards the upper bed interface. The upward movement of bubbles from the lower layers delays the collapse in the first stage and represents the bubbling period.

**Figure 5:**
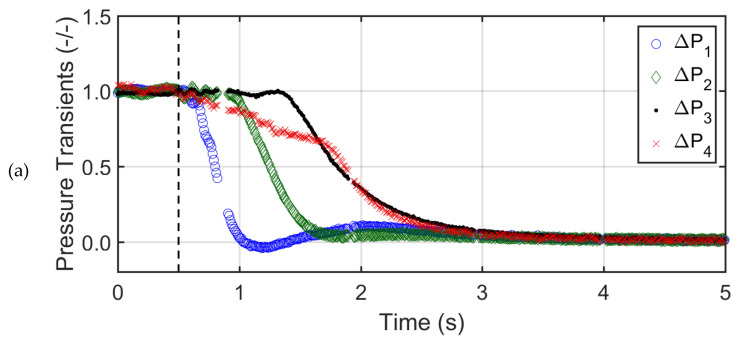

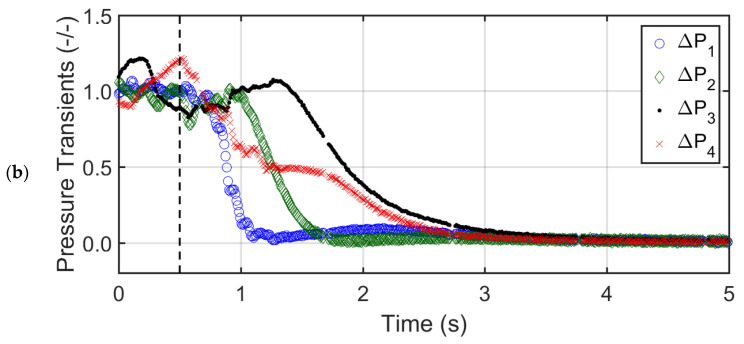
Pressure drop transients in different regions of the bed of glass beads for (**a**) $U_{0}=U_{\mathrm{mf}}$, (**b**) $U_{0}=2U_{\mathrm{mf}}$.

Figure 5a,b show the local bed pressure drop transients for glass beads at two different air superficial velocities ($U_{0}$), i.e., $U_{0}=U_{mf}$ and $U_{0}=2U_{mf}$. The dashed black vertical line indicates the commencement of the collapse process. To facilitate the comparison between different sections of the collapsing bed, the actual transient pressure drop data was normalized with respect to the steady state pressure drop before the start of the bed collapse. The upper sections of the bed displayed a delayed response to the collapse as compared to lower sections. This phenomenon was due to the movement of air bubbles during the first phase of the collapse process. The upper region transients ($∆P_{4}$) showed the longest bubbling period, which was also the total time taken by the bubbles to escape after the start of the collapse process. Moreover, the collapse behavior of the uppermost layer was different from the other layers, as the pressure transients decreased steadily during the bubbling phase due to the downward movement of the top bed interface. The sedimentation stage of the collapse process followed an exponential decay in all bed regions before attaining a complete rest. The sedimentation stage transients were similar for all the bed regions, thus indicating the bed homogeneity and uniform size of settling particles. As the initial air superficial velocity was increased to $2U_{mf}$, the fluctuations during the first phase increased due to the higher volume of air escaping as bubbles from the top interface of the glass bed.

**Figure 6:**
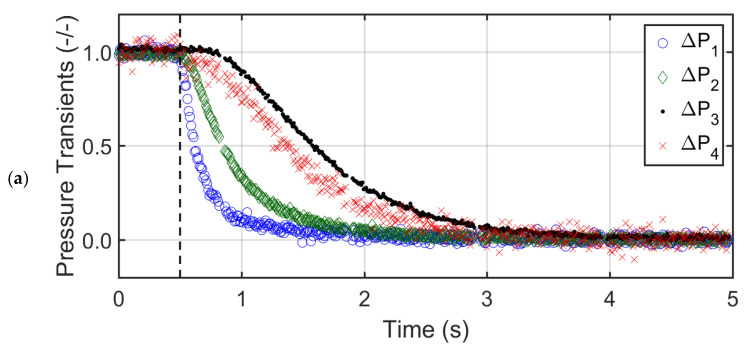

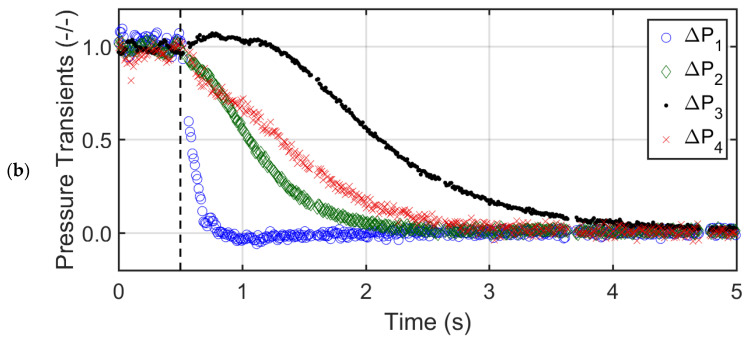
Pressure drop transients in different regions of the bed of titania powder for (**a**) $U_{0}=U_{\mathrm{mf}}$, (**b**) $U_{0}=2U_{\mathrm{mf}}$.

Figure 6a,b report the pressure transients of the collapsing bed of titania powder at $U_{0}=U_{mf}$ and $U_{0}=2U_{mf}$. The bubble escape time was significantly shorter (less than half) than that of the glass beads due to the smaller bubble concentration. The bubbling stage was observed only in $∆P_{3}$ transients with a bubbling time of approximately 0.3 s and 0.7 s for $U_{0}=U_{mf}$ and $2U_{mf}$, respectively. The $∆P_{3}$ region dominated the bed collapse process. The bubbling stage was not observed in the $∆P_{2}$ and $∆P_{4}$ transients, which could be attributed to the presence of cracks and channels in the upper and lower middle regions of the titania bed. Due to the poor contact between the gas and solid phases, the gas–solid drag and hence the pressure drop was low in the upper region of the bed during the collapse. As a result, the smaller agglomerates present in the upper middle layer mainly controlled the collapse process owing to size-based segregation. The size segregation of the agglomerates along the bed height also affected the sedimentation stage transients. 

Faster transients were observed for the lower layer that contained large agglomerates. $∆P_{1}$ transients were followed by that of $∆P_{2}$. Owing to the presence of fine agglomerates, the $∆P_{3}$ transients were slowest. The collapse behavior was more pronounced for $U_{0}=2U_{mf}$ (Figure 6b). Higher velocities increased the bubble fraction, thus enhancing the bubbling time for $∆P_{3}$ transients. The segregation behavior was also stronger at higher velocities, thereby slowing the pressure drop transients of the upper middle layer (i.e., $∆P_{3}$). The $∆P_{4}$ transients appeared unaffected by the velocity change as the uppermost layer apparently developed cavities or channels through which the fluidizing gas bypassed without much interaction with bed material.

**Figure 7:**
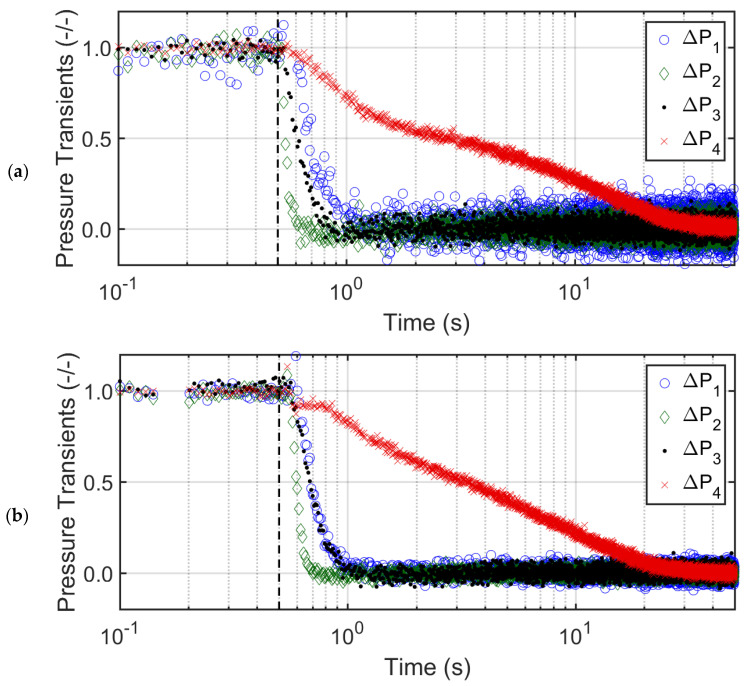
Pressure drop transients in different regions of the bed of nanosilica for (**a**) $U_{0}=U_{\mathrm{mf}}$, (**b**) $U_{0}=2U_{\mathrm{mf}}$.

Figure 7 highlights the behavior of the pressure transients in the nanosilica bed. In Figure 7a, the pressure transients, $∆P_{1}$, $∆P_{2}$, and $∆P_{3}$ responded instantaneously to the collapse, whereas $∆P_{4}$ transients decreased gradually taking approximately 25 s to settle completely. The static bed height was 0.4 m, which expanded to 0.7 m at $U_{\mathrm{mf}}$, thus almost half of the bed was in the $∆P_{4}$ region. The rapid decrease in $∆P_{1}$, $∆P_{2},$ and $∆P_{3}$ transients indicated a lack of agglomerate movement in the lower regions due to the size segregation in the bed. Large rigid agglomerates constituted the lower region whereas fine agglomerates were observed in the upper region. Clearly, the size segregation in the nanosilica bed was much higher than the titania bed. The $∆P_{4}$ transients indicated that the collapse process occurred in two stages. In the first stage, free fall of solids occurred under gravity with a rapid pressure drop decrease for approximately 1.5 s. During the second stage, the response was sluggish due to a gradual agglomerate settling and slow deaeration of the residual air through the bed and the purge.

**Figure 8:**
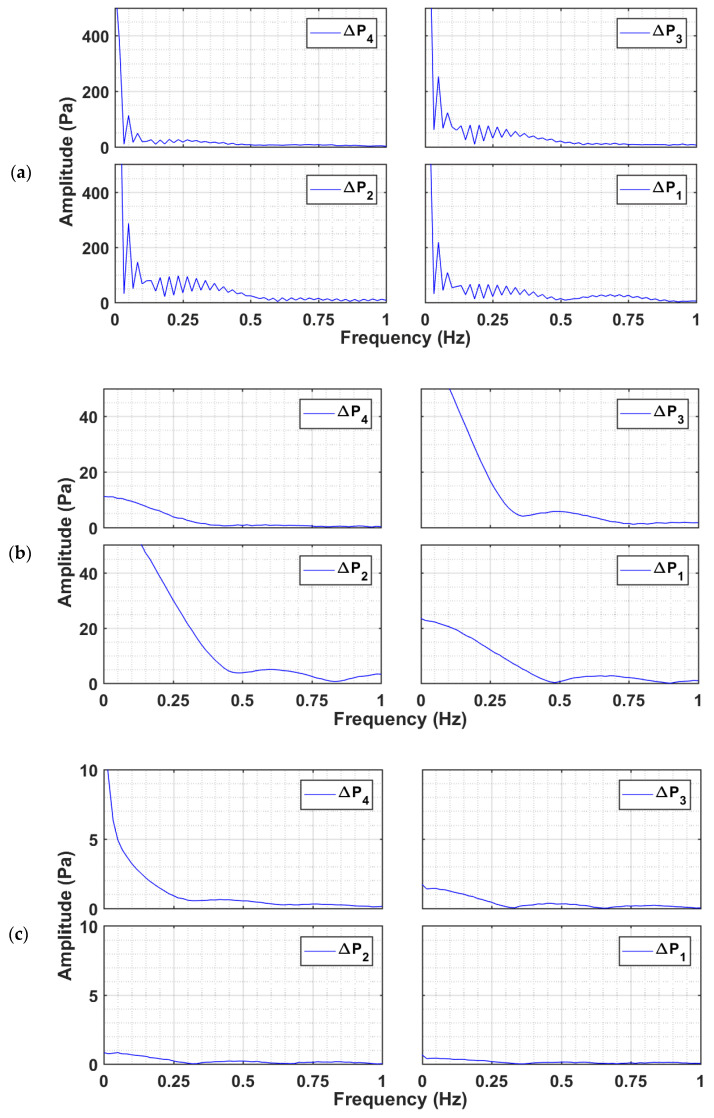
Power spectral analysis at low frequencies (0–1 Hz) of pressure transients in different bed regions of the collapsing bed at $U_{\mathrm{mf}}$ and bed height H of (**a**) glass beads, (**b**) titania, and (**c**) nanosilica.

The data analysis in the frequency domain can identify the presence of periodically recurring events in the bed, which cannot otherwise be clearly identified from the time domain data analysis [42]. Figure 8 reports the frequency spectra of the pressure drop transients recorded during the bed collapse at low frequencies using fast the Fourier transform (FFT) algorithm of MATLAB R2015a (Mathworks, Natick, MA, USA). For the case of the glass beads, the multiple low frequency peaks observed in Figure 8a indicate bubble movement. Given that the bed voidage of the glass beads at $U_{\mathrm{mf}}$ is approximately 0.38, its bulk density is high, leading to high pressure drop values in all the regions of the glass bed. This behavior is clearly evident from the dc component of the signal, which is <400 Pa in all the transients. The dc component refers to the amplitude of the non-fluctuating component of the signal, which is equal to the mean value of the signal. 

This situation is substantially different for the titania, with a significantly higher voidage (ε=0.80) and a lower bulk density than that of glass. Therefore, pressure drop values, i.e., the dc components of the signals are substantially lower than those of glass (Figure 8b). Ideally, the dc components of $∆P_{1}$, $∆P_{2}$, $∆P_{3}$ should be identical given that $∆L_{1}=∆L_{2}=∆L_{3}$. Therefore, the lower $∆P_{1}$ indicates the presence of gas bypassing due to the non-homogeneities in the lower layer of the titania bed. The same phenomenon was also prominently observed in the upper layer, where $∆P_{4}$ transients were recorded. No low frequency event was observed in this region unlike $∆P_{2}$ and $∆P_{3}$, where weak bubble movement with a frequency of 0.5 Hz was observed. Due to the local non-homogeneities, the bubble movement in the lower region appeared to be suppressed.

The spectral analysis corroborates the different bubbling phenomenon occurring in the glass and titania beds discussed earlier. The smaller and irregular gas bubbles were observed in the titania bed while homogeneous intense bubbling occurred throughout the bed of glass beads. In Figure 8c, no evidence of bubbling was noticed in the low frequency range for the nanosilica. However, the magnitude of the dc component, (i.e., pressure drop) was highest for $∆P_{4}$, which kept on decreasing for $∆P_{3}$ and $∆P_{2}$ while being the lowest for $∆P_{1}$. The large and rigid agglomerates in the lower regions behaved like a fixed bed, where the pressure drop varied inversely with the agglomerate size as predicted by the Ergun equation. Therefore, the gradually increasing dc component along the bed height (Figure 8c) indicated that the size of the agglomerates decreased with the distance from the distributor, thereby leading to the size-based segregation of agglomerates. The upper region of the nanosilica bed is clearly seen to contribute the most to the pressure drop.

**Figure 9:**
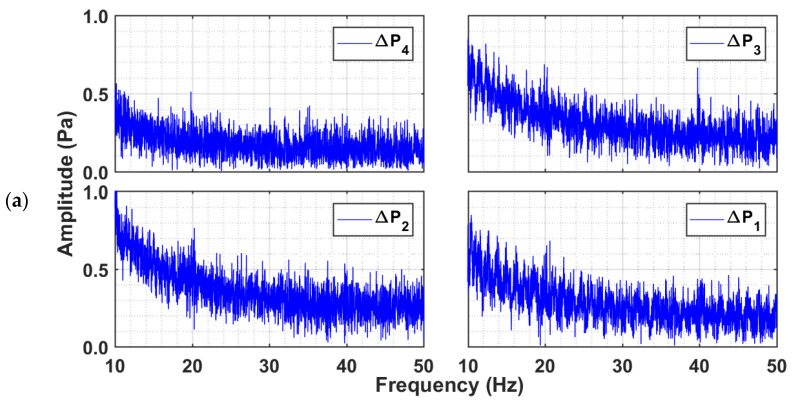

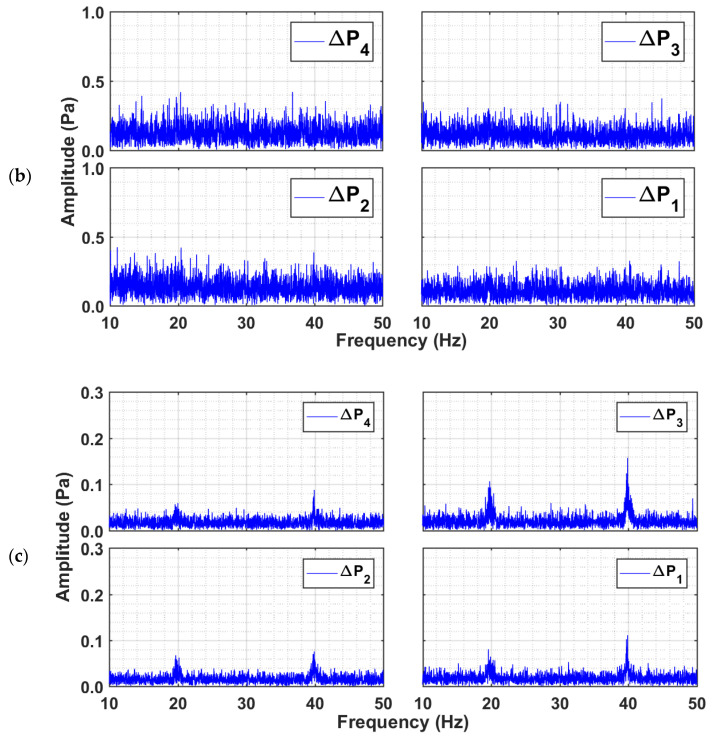
Power spectral analysis at low frequencies (10–50 Hz) of pressure transients in different bed regions of the collapsing bed at $U_{\mathrm{mf}}$ and bed height H of (**a**) glass beads, (**b**) titania, and (**c**) nanosilica.

Figure 9 reports the power spectra at higher frequencies (10–50 Hz) for all the three powders. No high frequency events were observed for the case of the glass and titania. However, the nanosilica shows two distinct peaks at 20 Hz and 40 Hz in Figure 9c, which clearly indicate the presence of high frequency events. The highest intensity fluctuation occurred in the $∆P_{3}$ transients. The feeble peaks in the $∆P_{4}$ region indicate lower intensity fluctuations caused by fine agglomerates present in the upper region due to segregation. The presence of high intensity fluctuations was clearly observed in Figure 7a, which were predominantly present during the sedimentation stage for regions with large agglomerates, while the fine and smaller agglomerates in the upper region were still settling under the gravity. The interaction of the larger agglomerates with their smaller counterparts appeared to the main reason for the high frequency events observed for the nanosilica.

**Figure 10:**
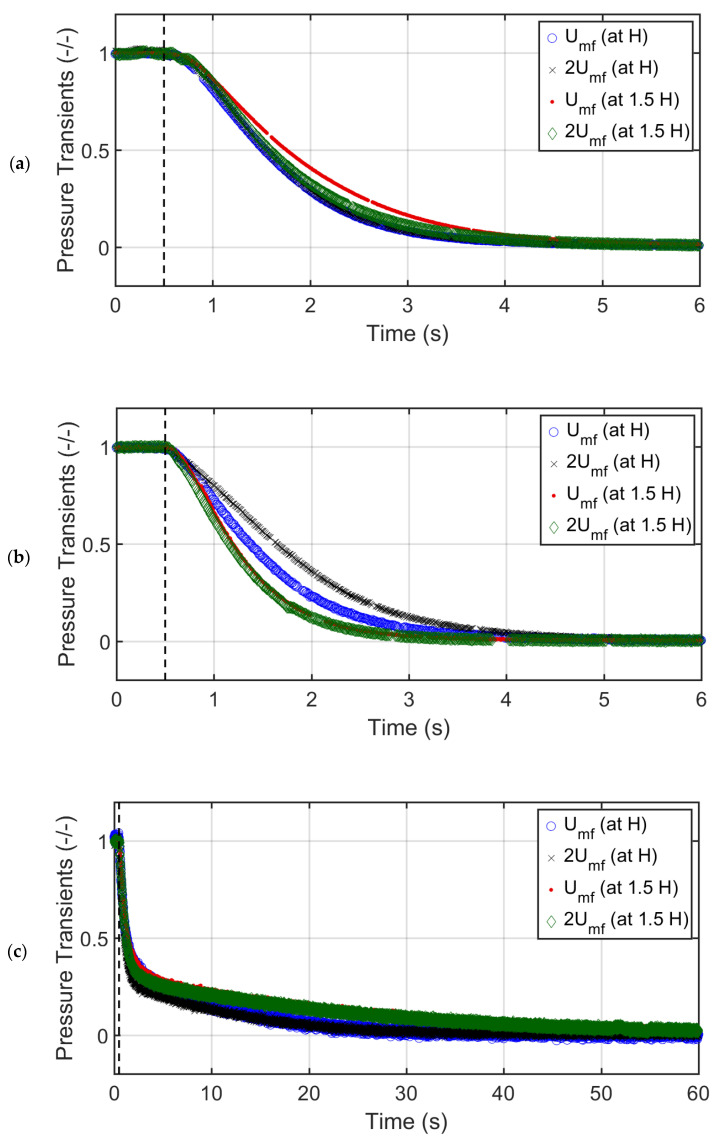
Global pressure drop of the collapsing particles bed at $U_{\mathrm{mf}}$ and $2U_{\mathrm{mf}}$ bed heights H and 1.5 H for (**a**) glass beads, (**b**) titania, and (**c**) nanosilica.

Figure 10 shows the effect of the height and the velocity on the global bed pressure drop transients for the beds of glass beads, titania, and nanosilica. Apart from the velocity, the effect of height on the bed dynamics was also considered by increasing the bed height by 50%, represented as 1.5 H in the figure. The global pressure drop included the combined effect of all the bed regions. A smooth overall collapse was clearly seen for all the cases. A substantial change in the velocity ($U_{0}=U_{mf}$ and $U_{0}=2U_{mf}$) failed to make any significant difference on the collapse time irrespective of the bed material used. Similar observations were made earlier by Lorences, Patience [26]. However, increasing the height by 50% affected the collapse time in all cases. In the case of the titania, the shorter beds were slower than the longer beds. Moreover, shorter titania beds were more sensitive to the initial superficial velocity at which the collapse was initiated. While the increase in the bed height did not significantly affect the collapse time for the glass beds, a 50% increase in the height increased the collapse time for the nanosilica by approximately 100%.

Table 2 presents a comparison of the collapse times for all three cases. The collapse time was much higher for nanosilica as compared to those of glass beads and titania, with similar collapse times. The collapse times for glass beads, titania, and nanosilica varied from 4.7–6.6 s, 4.1–7.1 s, and 22.3–43.6 s, respectively. As mentioned earlier, unlike other cases, the bed height significantly affected the collapse time of the nanosilica. A 50% increase in the bed height led to an almost 100% increase in the collapse time for nanosilica. Adding to the bed material to increase the initial bed height led to a higher concentration of fine agglomerates in the upper region, which attained greater height in the column owing to bed expansion. These fine agglomerates took much longer to settle during collapse. Surprisingly, the trend was reversed for the titania bed. Adding to the bed material shortened the collapse time. This is a clear indication that increasing the bed material did not correspondingly increase the gas–solid contact. Instead, greater bed non-homogeneity in the bed developed, thereby reducing the gas–solid drag. As a result, the collapse time was reduced.

### 3.3. Distributor Pressure Drop

**Figure 11:**
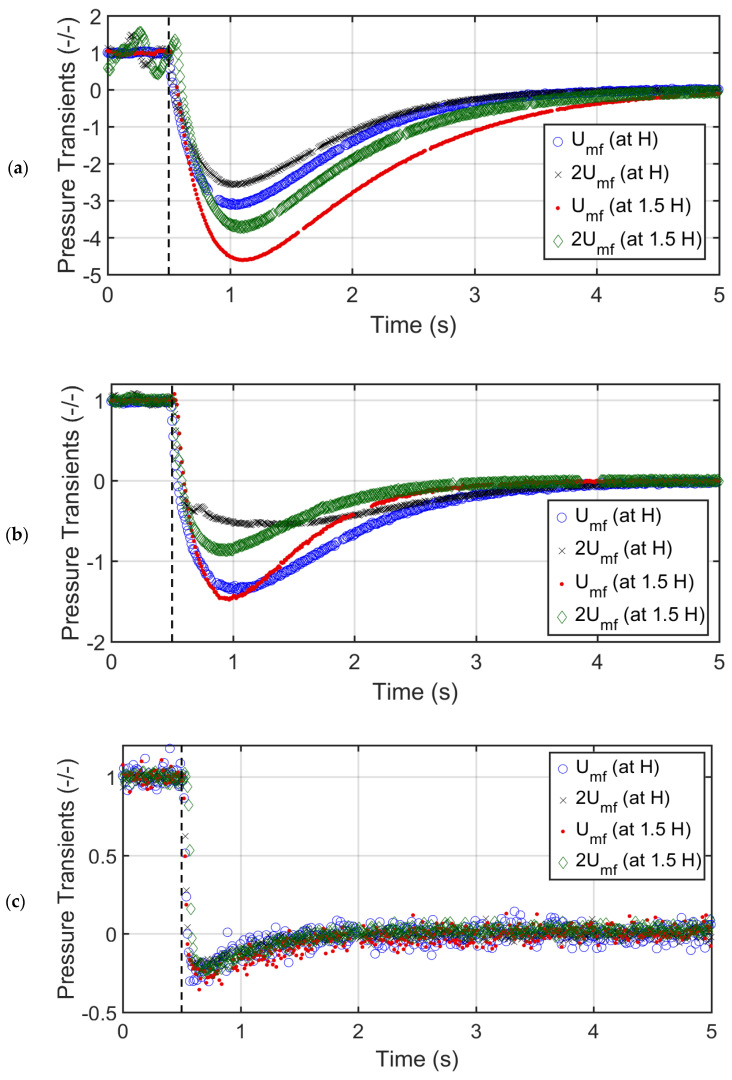
Distributor pressure drop of the bed at $U_{\mathrm{mf}}$ and $2U_{\mathrm{mf}}$ for (**a**) glass beads, (**b**) titania, (**c**) nanosilica.

An important aspect of the present investigation was the monitoring of the distributor pressure drop during the bed collapse. The distributor pressure drop was mostly higher than the bed pressure drop given that only 2.7% of the distributor area was kept open to gas flow. Figure 11a reports the effect of the bed height and the velocity on the distributor pressure drop during the collapse of the glass bed. As the collapse process commenced, the distributor pressure drop decreased rapidly. A resultant net drag force was exerted downwards by the falling particles, which forced a portion of residual air present in the column to flow in the reverse direction across the distributor, which resulted in a rapid decrease of the pressure drop. The pressure drop became negative until reaching a global minimum before gradually rising back to zero pressure drop, while the collapsing bed reached a complete rest. The opening of the purge valve (SV2) during the collapse process provided an alternative path for residual air in the reverse direction in addition to the conventional pathway from the top of the bed. A higher initial collapse velocity resulted in a higher negative pressure drop because the bed contained a greater volume of air at higher velocities. Increasing the bed height by 50% increased the negative minimum by approximately 50% irrespective of the velocity, owing to bed homogeneity and uniform expansion.

Figure 11b reports the distributor pressure drop transients during the collapse of the titania bed. The effect of the bed height was not significant at a lower velocity as the gas bypassing occurred through channels and cracks. As a result, increasing bed solids did not increase the gas–solid drag that can cause a greater downward flow of residual air during the collapse. However, at a higher velocity, there was a substantial difference in the distributor pressure drop transients when the bed height was changed. Faster transients were observed for the longer beds as compared to those of the shorter beds.

For the case of the nanosilica particles shown in Figure 11c, the effect of the velocity and the bed height on the distributor pressure drop transients was negligible. Given that the voidage was 0.98, only 2% solids were present in the bed. Due to the extremely high bed voidage, any change either in the velocity or the bed material failed to make any difference on the deaeration of the residual gas from the nanosilica bed. 

Figure 11 indicates that the bed voidage clearly affected the pressure drop minimum. The maximum negative pressure drop values attained by the glass beads, titania, and nanosilica collapse beds were approximately −4.7, −1.5 and −0.65, respectively. A low bed voidage leads to a higher specific surface area of the solid phase in the bed, resulting in greater gas–solid drag and frictional losses during the bed collapse which forces a greater amount of the residual air to pass through the distributor.

### 3.4. Purge Flow Transients

**Figure 12:**
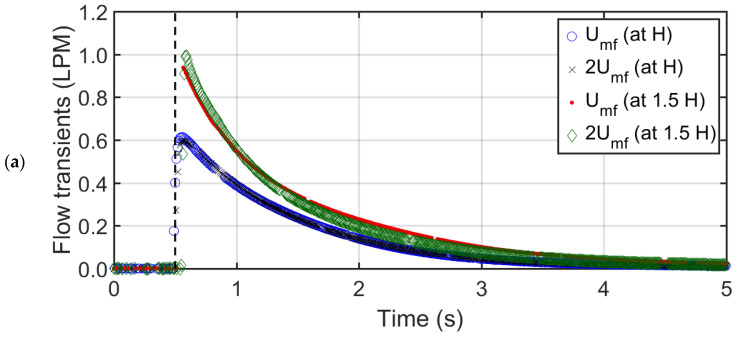

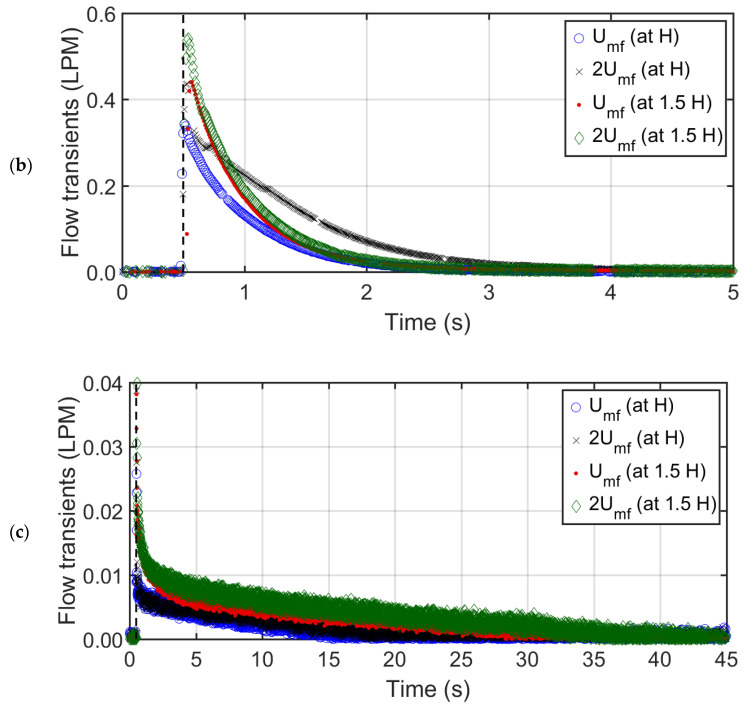
Purge flow transients at $U_{\mathrm{mf}}$ and $2U_{\mathrm{mf}}$ for (**a**) glass beads, (**b**) titania, and (**c**) nanosilica.

Figure 12 reports the airflow transients in LPM through the purge valve during the collapse process of glass beads, titania, and nanosilica particles bed. The purge flow begun with a sudden airflow spike as the collapse initiated, followed by a sluggish exponential decay. Table 3 reports the maximum airflow from the purge, recorded from Figure 12, achieved during the commencement of collapse. The lower voidage bed attained higher peak. The highest peaks attained by glass beads, titania, and nanosilica particles were 0.99, 0.54, and 0.02 LPM, respectively. The initial spike signifies the initial intensity of the bed collapse. The particles with a higher bed weight and uniform fluidization forced the downward flow of the residual air through the purge valve. Moreover, increasing the initial airflow from $U_{mf}$ to $2U_{mf}$ did not yield any tangible effect on the purge flow. However, increasing the bed height resulted in an increase in the higher flow spike in all cases, which signifies the flow spike was dependent on the fluidized bed weight. These parameters are reported in Table 3.

**Figure 13:**
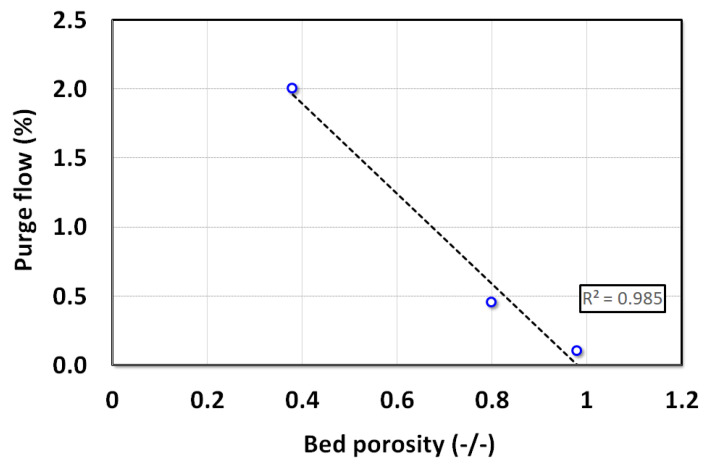
Effect of the bed voidage on the ratio of the purge flow to the total volume during gas deaeration.

The total airflow in LPM, which is the total area under the curve in Figure 12, was calculated using the trapezoid method. Thereafter, the ratio of the total airflow through purge to the volume of air that was present in the bed during the fluidization before the commencement of the collapse was calculated. The volume of air present in the bed resides in the voidage of the bed. Hence, bed voidage volume gives a good approximation of the total volume of air present under steady state. Figure 13 reports the plot of purge airflow ratio against the bed voidage. The relationship is linear with ‘R^2^ = 0.985’ fit, which signifies that the ratio of purge airflow with bed fluid volume during fluidization decreases linearly with the bed voidage.

## 4. Conclusions

The bed collapse behavior of three different kinds of solid particles of widely different voidages and belonging to different Geldart classifications, namely glass beads, titania, and nanosilica, was investigated in this study. First, the hysteresis behavior and fluidization index of each solid particle species was examined by analyzing the dependence of the pressure drop on the air velocity while both gradually increasing and decreasing the air flow. The glass beads (Geldart group A) showed the highest fluidization index of 0.92 with no hysteresis effects. The cohesive powder of titania (Geldart group C) showed severe gas-bypassing due to local non-homogeneities comprising cracks and channels, thereby resulting in a low fluidization index of 0.76 and a strong hysteresis effect. The fluidization index of nanosilica was even lower at 0.61 due its poor fluidization hydrodynamics with a stronger hysteresis behavior than that observed for the titania. 

A dual drainage scheme was implemented by allowing the downward movement of the residual air through a purge passage located below the plenum chamber. The flowrate of the exiting air through the purge was monitored using an electronic flowmeter. Owing to the bed homogeneity, all the regions in the glass bed exhibited almost identical two-stage collapse processes, i.e., the bubbling and sedimentation stages. The bubbling time decreased with the distance from the distributor. The spectral analysis of the transients clearly indicated the bubble movement as low frequency events. The bubbling was less intense and irregular due to the gas-bypassing through the cracks and channels in the bed of the titania particles. Its transient response decreased with the distance from the distributor owing to size segregation with the upper middle layer (i.e., $∆P_{3}$) dominating the collapse dynamics. Even stronger segregation tendencies were observed in the nanosilica bed with a significant number of fine agglomerates present in the upper region of the bed while large rigid agglomerates occupied the lower region. Therefore, the slow gravity settling of fine agglomerates in the upper region took almost 25 s whereas larger agglomerates present in the other bed regions settled in approximately 1 s only. As a result, the nanosilica bed collapse took at least five times longer than the durations of glass and titania, which showed almost identical overall collapse times. Unlike glass and titania, two distinct high frequency events at 20 Hz and 40 Hz occurred for nanosilica due to the interaction of fine agglomerates with their larger counterpart while setting down.

For glass beads, the maximum negative distributor pressure drop attained during the collapse was a function of both the velocity and bed height. The same behavior was observed with the titania bed with a change of the bed height. For the case of the nanosilica, however, the backward flow of the residual air through the distributor was rather insignificant, resulting in a low pressure drop.

Hydrophilic nanosilica with strong agglomeration behavior were used in this study. Any extension of these results to nanoparticles with APF behavior, even with similar primary dimensions and comparable bulk density, should be treated with caution.

## Figures and Tables

**Table 1:** Physical properties of solid particles used in the experiments.

Powder	Particle Density (kg/m^3^)	Particle Size (×10^−6^ m)	Bulk Density (kg/m^3^)	Bed Voidage(-/-)	*U_mf_* (×10^−3^ m/s)
Glass beads	2500	88–149	1547	0.38	31
TiO_2_	3900	0.5–90	790	0.80	19
Nanosilica [26,39]	2200	2–100	50	0.98	30

**Table 2:** Total bed collapse time (in seconds) evaluated using global pressure drop transients.

Static Bed Height	H		1.5 H	
Initial Flow Rate	*U_mf_*	2*U_mf_*	*U_mf_*	2*U_mf_*
Glass beads	5.0	4.7	6.6	5.8
Titania	5.7	7.1	4.2	4.1
Nanosilica	22.3	25.7	43.6	43.5

**Table 3:** Maximum initial airflow (LPM) through purge during collapse evaluated from Figure 12.

Static Bed Height	H		1.5 H	
Initial Velocities	*U_mf_*	2*U_mf_*	*U_mf_*	2*U_mf_*
Glass beads	0.64	0.61	0.94	0.99
TiO_2_	0.34	0.44	0.41	0.54
Nanosilica	0.01	0.01	0.014	0.016

## Data Availability

The data that support the findings of this study are available on request from the first author, SSA.
